# Rethinking biologic and pregnancy research: The importance of assessing postpartum immunosuppression of the infant

**DOI:** 10.1016/j.ijwd.2021.06.007

**Published:** 2021-07-03

**Authors:** Mary Kathryn Abel, Alyssa Gwen Ashbaugh, Jenny E. Murase

**Affiliations:** a Department of Dermatology, University of California, San Francisco, San Francisco, California; b Department of Dermatology, University of California, Irvine, Irvine, California; c Department of Dermatology, Palo Alto Foundation Medical Group, Mountain View, California

**Keywords:** Biologics, pregnancy, biologics in pregnancy

Dear Editors,

We read with interest the publication by Kimball et al. (2021) entitled “Update on biologic safety for patients on biologic therapy in pregnancy,” published in the *International Journal of Women's Dermatology*. Indeed, biologic antipsoriasis medications are increasingly recognized as safe during pregnancy; however, most often the analysis is retrospective, and the majority of the research is in rheumatology patient populations and examines rates of developmental anomalies and birth outcomes (Porter et al., 2017). Kimball et al. (2021) recently published results from a cohort study evaluating pregnancy outcomes of patients receiving treatment for moderate-to-severe psoriasis with biologic or conventional systemic therapies. The authors’ findings corroborated what was previously published in the *International Journal of Women's Dermatology* and provide further reassurance that pregnant women treated with these medications had overall pregnancy and birth outcomes, including congenital anomalies, spontaneous abortions, and live birth, preterm, and stillbirth rates, similar to those in the general population. This excellent report uses one of the largest cohorts of patients with psoriasis and provides reassurance that biologic and systemic treatments for psoriasis are safe during pregnancy.

Infliximab and other antitumor necrosis factor alpha agents are the most commonly prescribed biologic therapies for psoriasis treatment and hold a pregnancy category B drug label by the U.S. Food and Drug Administration (Murase et al., 2014). Any maternal antibody that requires transport by the neonatal FC receptor, including monoclonal IgG antibodies such as infliximab and adalimumab, would not begin to cross the placenta until mid-second trimester. The majority of organ development occurs during the first trimester, so the lack of increase in congenital anomalies and spontaneous abortion with maternal use of these biologics corroborates Kimball et al.’s findings. The passage of antibodies to the fetus does exponentially increase in late third trimester when there is active transport across the placenta and the fetus's immune system is primed by the transfer of these maternal antibodies; in fact, cord blood levels of infliximab and adalimumab were much higher (160% and 153%, respectively) than that of maternal blood (Mervic, 2014).

These antibodies are known to persist in the infant's serum for 2 to 7 months after birth (Esteve-Solé et al., 2017). As documented by prior case reports, these infants may be subsequently immunosuppressed after birth and unable to develop appropriate immune responses to vaccines or infections, as demonstrated by an infant death after the administration of a live vaccine at 3 months of life (Cheent et al., 2010; Heller et al., 2011). Thus, the essential question regarding the use of biologic psoriasis therapy during pregnancy is not what happens in utero, but rather what the degree of immunosuppression the infant experiences within the first few months of life. This has not been studied in the infants of patients with psoriasis. Cohorts of children whose mothers took cyclosporine, another systemic therapy for severe psoriasis, during pregnancy have been followed through early childhood and revealed no detectable long-term neurodevelopmental, immunologic, and nephrotoxic effects. As such, future studies are necessary to assess the effect on the immune system of children exposed to biologic psoriasis agents in utero, similar to those performed in the transplant literature for cyclosporine (Cochat et al., 2004; Nulman et al., 2010; Shaheen et al., 1993).

In this vein, patients who receive certolizumab and their offspring should be analyzed separately in this and future research. Certolizumab is a common treatment for moderate-to-severe psoriasis that does not cross the placental barrier because it is nonpegylated and does not bind the neonatal FC receptor (Ferreira et al., 2020). Thus, the offspring of these patients are not subjected to the same level of immunosuppression as those receiving anti-TNF-α agents. The offspring of this cohort would be a fascinating comparison group, particularly when studying the immunologic outcomes of infants during the first year of life exposed to antipsoriasis biologic medications.

Kimball et al.’s findings reaffirm the safety of psoriasis treatments during pregnancy itself and the likelihood that there is no increased risk of developmental defects as a result of in utero biologic exposure during pregnancy, as was initially hypothesized (Carter et al., 2006). However, the concern with biologic agents is more of a third trimester issue of antibody transfer and subsequent infantile immunosuppression than a first trimester issue of developmental anomaly risk. The impact of the antibody boost that the infant receives immediately before delivery and the subsequent potential for increased immunosuppression if these antibodies are immunosuppressive biologic agents should not be ignored and should be further investigated to truly provide reassurance of the safety of biologic therapy during pregnancy. Future parents deserve to know the degree to which nonpegylated antipsoriasis biologic medications will suppress their child's immune system. As providers, we cannot answer this question until further work is done.

## References

[bib0001] Carter JD, Valeriano J, Vasey FB. (2006). Tumor necrosis factor-α inhibition and VATER association: A causal relationship?. J Rheumatol.

[bib0002] Cheent K, Nolan J, Shariq S, Kiho L, Pal A, Arnold J. (2010). Case report: Fatal case of disseminated BCG infection in an infant born to a mother taking infliximab for Crohn's disease. J Crohn's Colitis.

[bib0003] Cochat P, Decramer S, Dubourg L, Audra P (2004). Renal outcome of children exposed to cyclosporine in utero.

[bib0004] Esteve-Solé A, Deyà-Martínez À, Teixidó I, Ricart E, Gompertz M, Torradeflot M (2017). Immunological changes in blood of newborns exposed to anti-TNF-α during pregnancy. Front Immunol.

[bib0005] Ferreira C, Azevedo A, Nogueira M, Torres T. (2020). Management of psoriasis in pregnancy – A review of the evidence to date. Drugs Context.

[bib0006] Heller MM, Wu JJ, Murase JE. (2011). Fatal case of disseminated BCG infection after vaccination of an infant with in utero exposure to infliximab. J Am Acad Dermatol.

[bib0007] Kimball AB, Guenther L, Kalia S, De Jong EMGJ, Lafferty KP, Chen DY (2021). Pregnancy outcomes in women with moderate-to-severe psoriasis from the Psoriasis Longitudinal Assessment and Registry (PSOLAR). JAMA Dermatol.

[bib0008] Mervic L. (2014). Management of moderate-to-severe plaque psoriasis in pregnancy and lactation in the era of biologics. Acta Dermatovenerol Alp Pannonica Adriat.

[bib0009] Murase JE, Heller MM, Butler DC. (2014). Safety of dermatologic medications in pregnancy and lactation: Part I. Pregnancy. J Am Acad Dermatol.

[bib0010] Nulman I, Sgro M, Barrera M, Chitayat D, Cairney J, Koren G. (2010). Long-term neurodevelopment of children exposed in utero to ciclosporin after maternal renal transplant. Pediatr Drugs.

[bib0011] Porter ML, Lockwood SJ, Kimball AB. (2017). Update on biologic safety for patients with psoriasis during pregnancy. Int J Womens Dermatology.

[bib0012] Shaheen FA, Al-Sulaiman MH, Al-Khader AA (1993). Long-term nephrotoxicity after exposure to cyclosporine in utero. Transplantation.

